# Achacha (*Garcinia humilis*) Rind Improves Cardiovascular Function in Rats with Diet-Induced Metabolic Syndrome

**DOI:** 10.3390/nu10101425

**Published:** 2018-10-04

**Authors:** Oliver D. John, Stephen Wanyonyi, Peter Mouatt, Sunil K. Panchal, Lindsay Brown

**Affiliations:** 1 Functional Foods Research Group, University of Southern Queensland, Toowoomba, QLD 4350, Australia; Oliver.John@usq.edu.au (O.D.J.); Stephen.Wanyonyi@usq.edu.au (S.W.); Sunil.Panchal@usq.edu.au (S.K.P.); 2 School of Health and Wellbeing, University of Southern Queensland, Toowoomba, QLD 4350, Australia; 3 Analytical Research Laboratory, Southern Cross Plant Science, Southern Cross University, Lismore, NSW 2480, Australia; Peter.Mouatt@scu.edu.au

**Keywords:** metabolic syndrome, obesity, inflammation, *Garcinia humilis*, blood pressure, procyanidin, flavonoids

## Abstract

*Garcinia humilis* is a fruit known as achachairú. It is native to South American countries such as Bolivia, Peru, and Brazil, but it is also cultivated as achacha in northern Australia. The aim of this study was to determine the phytochemicals in achacha rind and pulp and to investigate these components as potential treatments for the symptoms of metabolic syndrome. Both rind and pulp contain procyanidins and citric acid rather than hydroxycitric acid. Male Wistar rats (8–9 weeks old) were fed with either high-carbohydrate, high-fat, or corn starch diets for 16 weeks. Intervention groups were fed with either diet supplemented with 1.5% *G. humilis* rind powder or 2.0% *G. humilis* pulp for the last 8 weeks of the protocol. Rats fed a high-carbohydrate, high-fat diet exhibited hypertension, dyslipidemia, central obesity, impaired glucose tolerance, and non-alcoholic fatty liver disease. *G. humilis* rind decreased systolic blood pressure, diastolic stiffness, left ventricular inflammatory cell infiltration, and collagen deposition in high-carbohydrate, high-fat diet-fed rats. However, there was no change in glucose tolerance, body weight, or body composition. Therefore, *G. humilis* rind, usually a food by-product, but not the edible pulp, showed potential cardioprotection with minimal metabolic changes in a rat model of diet-induced metabolic syndrome.

## 1. Introduction

The more than 300 *Garcinia* species belonging to the *Clusiaceae* family are a potential source of medicinal phytochemicals [1,2]. *Garcinia humilis*, also known as *Garcinia achachairú* [3], *Mammea humilis* [3], *Rheedia lateriflora* [4], *Rheedia sessiliflora*, and *Rheedia sieberi* [3], is distributed widely in Brazil and eastern Bolivia [5], and also found in Peru, Guatemala, Guyana, Panama, and the Caribbean Islands [3]. The fruit has been traditionally used as a hunger suppressant [6] and for healing the skin [3]. The rind can be made into a drink by blending and overnight infusion in water [4,6], and the pulp has a flavor resembling mangosteen [4]. It was pioneered commercially in North Queensland, Australia, with the first fruit on the market in 2012, followed by the initial commercial Guatemalan harvest in 2018 [6]. The reported medicinal properties of *G. humilis* include gastroprotective properties in ethanol/HCl-induced gastric lesions in mice [7,8], antinociceptive effects [7], leshmanicidal effects [9], and antiproliferative activity [10].

*Garcinia* fruits contain organic acids such as hydroxycitric acid [11]. They also contain many oxygenated and prenylated xanthones including prenylated benzophenones, such as guttiferones and bioflavonoids, with pharmaceutical and biological properties [7,12]. Benzophenones are the key intermediates in the biosynthetic pathway of xanthones [13]. So far, there have been no reports on the phytochemical analysis of the rind and pulp of *G. humilis*.

Metabolic syndrome leads to a cluster of risk factors such as dyslipidemia, insulin resistance, central obesity, and hypertension that collectively increase the risk of obesity, type 2 diabetes, and cardiovascular disease [14,15,16]. Obesity is generally linked with excess energy intake and low levels of physical activity [16,17]. Consumption of the Western diet increases the intake of refined sugars and saturated fats [18], which has been associated with the development of metabolic syndrome [18,19]. In contrast, increased intake of fruits, vegetables, cereals, and legumes could reduce metabolic syndrome markers [20]. Treatments for metabolic syndrome generally involve lifestyle and pharmacological interventions to target the individual symptoms [16].

In this study, we evaluated the liver, cardiovascular, and metabolic responses to *G. humilis* rind and pulp by using an established high-carbohydrate, high-fat diet model mimicking the human metabolic syndrome [21]. Measurements included body weight, systolic blood pressure, oral glucose tolerance, left ventricular collagen deposition and stiffness, histology of the liver, organ weights including abdominal fat, and plasma biochemistry. Our hypothesis was that regular consumption of achacha fruit would reverse metabolic, cardiovascular, and liver changes in diet-induced metabolic syndrome.

## 2. Materials and Methods

### 2.1. G. humilis Rind and Pulp Powder Preparation and Analyses

*G. humilis* fruits were obtained from Achacha Fruit Group, Giru, Queensland. The fruits were then separated into rind, pulp, and seed, and weighed. The rind and pulp were stored at −20 °C, freeze-dried, and ground into powder. Samples of rind and pulp powder were analyzed to detect the compounds present. The remaining powder was kept at 4 °C until further use.

The quantification of procyanidins in the sample was performed by high-performance liquid chromatography (HPLC) using a method developed for analytical purposes based on previously published methods using hydrolysis and derivatization of the flavanol units to quantify the varying polymeric forms of procyanidins [22]. Briefly, hydrolysis of the B-type procyanidins, with single bonds between each flavanol unit, in the presence of the nucleophile phloroglucinol, forms reaction products with each flavanol unit except the terminal unit from the procyanidin. The range of polymeric procyanidins is reduced to a series of common products for quantification calculated as the products of reference dimeric procyanidin B2.

The hydrolysis reagent solution consisted of 0.2 M HCl containing 10 mg/mL phloroglucinol and 10 mg/mL ascorbic acid. A 160 mM solution of sodium acetate was prepared for quenching the hydrolysis. Briefly, dried samples were extracted into 50:50 ethanol:water solution with sonication for 15 minutes. One aliquot was taken and diluted 0.5 mL into 7.5 mL with 50:50 ethanol:water solution to determine free flavanol content. Another 0.5 mL aliquot was placed with 2 mL of phloroglucinol reagent in a reaction tube and heated in a water bath at 78 °C for 60 minutes. After cooling, 5 mL of the sodium acetate solution was added to quench the hydrolysis reaction, with a final volume of 7.5 mL. Aliquots of both the diluted and hydrolyzed samples were placed in a mini-vial for HPLC analysis. For quantification, a catechin reference standard was prepared as a serial dilution from 0.01 to 1 mg/mL in 50:50 ethanol:water solution. Dimeric procyanidin B2 (PhytoLab, Vestenbergsgreuth, Germany) was also prepared as a serial dilution from 5 to 0.05 mg/mL; then 0.5 mL aliquots were taken and diluted or hydrolyzed with the phloroglucinol (Sigma-Aldrich Australia, Castle Hill, NSW, Australia) reagent as per the test sample. A calibration curve of the hydrolysis products of procyanidin B2 was used to calculate the procyanidin content of the test sample. Free flavanols were calculated using catechin for the unhydrolyzed sample and total procyanidins were calculated from the hydrolyzed sample.

Analysis of organic acids was based on the 2016 USP *Garcinia* hydroxycitric acid method. All reagents and solvents used were HPLC grade with Milli-Q water. Mobile phase was 0.136% (w/v) potassium dihydrogen phosphate in 3% phosphoric acid (Sigma-Aldrich Australia) adjusted to pH 2.5. Briefly, about 250 mg of the extracts were weighed and extracted in 5 mL of 3% phosphoric acid with sonication for 15 minutes. After centrifuging for 5 minutes, an aliquot of the supernatant was taken up in a HPLC vial and run against reference standards of hydroxycitric acid calcium salt (ChromaDex, Irvine, CA, USA), citric acid (Sigma-Aldrich Australia), and malic acids (Sigma-Aldrich Australia).

The analyses were performed on Agilent 1200 series HPLC, using a Phenomenex 250 mm C18 column with 1 mL/minute of isocratic mobile phase over 25 minutes. Reference standards were injected and prepared as a calibration curve for calculation of the hydroxycitric and citric acid concentrations. For the hydroxycitric acid profile, the rind of *Garcinia quaesita* was held as a reference sample at Analytical Research Laboratory for herbal authentication.

The dried extracts were analyzed by liquid chromatography-mass spectrometry (LC-MS) using an Agilent 1200 HPLC system with diode array detection (DAD) from 190 to 800 nm coupled with an Agilent single quadrupole mass spectrometer. The mass spectral analysis was performed with atmospheric pressure chemical ionization (APCI) operated in positive mode. The chromatography was performed on a Phenomenex luna C18 HPLC column (100 × 4.6 mm) using Milli-Q water and acetonitrile (Scharlau supragradient HPLC grade) with 0.005% trifluoroacetic acid (Sigma-Aldrich Australia). The method involved a gradient of 10% acetonitrile, 90% water to 95% acetonitrile, and 5% water over 18 minutes, at a flow rate of 0.75 mL/minute.

Peaks were tentatively identified based on UV-visible and mass spectra, in comparison with literature reports and previous phytochemical analysis, and, where possible, by comparison with reference standards such as catechin (Sigma-Aldrich Australia).

### 2.2. Rats and Diets

The experimental group comprised of 72 male Wistar rats (8–9 weeks old) supplied by the Animal Resource Centre, Murdoch, WA, Australia, and housed individually in a temperature-controlled room (22 ± 2 °C) under a 12-h light/dark cycle environment with free access to food and water at the University of Southern Queensland animal house. All experimental protocols were approved by the University of Southern Queensland Animal Ethics Committee (Project number: 15REA001), which operates under the guidelines of the Australian National Health and Medical Research Council. Rats were acclimatized for a week and upon reaching 335 ± 2 g body weight, they were randomly divided into 6 experimental diet groups (*n* = 12/group) and fed with corn starch (C), corn starch mixed with 1.5% *G. humilis* (achacha) rind powder (CAR), corn starch mixed with 2.0% achacha pulp powder (CAP), a high-carbohydrate, high-fat diet (H), a high-carbohydrate, high-fat diet mixed with 1.5% achacha rind powder (HAR), and a high-carbohydrate, high-fat diet mixed with 2.0% achacha pulp powder (HAP). As examples, 1 kg of food contains 15 g of rind powder (CAR or HAR) or 20 g of pulp powder (CAP, HAP). The pulp contains only about 5% of the procyanidin B2 concentration of the rind, so we adjusted the dose of rind to 1.5% of the diet to provide ~50 mg/kg/day of procyanidin B2 for comparison with literature studies. Treatment foods were given to CAR, CAP, HAR, and HAP groups for the last 8 weeks of protocol while C and H rats continued receiving the control diet during the whole 16-week protocol. The composition of the C and H diets has been reported [21]. The H, HAR, and HAP rats were given 25% (w/v) fructose in their drinking water for 16 weeks. Measurements of body weight, food, and water intakes were recorded daily and feed efficiency was calculated [21]. Daily energy intake was calculated from the daily food and water intakes from week 8 to week 16 [21]. Increase in body weight (%) was the difference in body weight between week 8 and week 16.

### 2.3. Rat Measurements

Oral glucose tolerance tests were performed on rats after overnight (12 h) food deprivation at 16 weeks; the fructose water in H, HAR, and HAP groups was replaced with normal water. Basal blood glucose concentrations were determined in tail vein blood using a Medisense precision Q.I.D. glucometer (Abbot Laboratories, Bedford, MA, USA). The rats were then given 2 g/kg body weight of glucose as 40% aqueous solution via oral gavage. Following this, blood glucose concentrations were measured at 30, 60, 90, and 120 minutes after glucose loading on tail vein blood samples [21].

Systolic blood pressure was measured at 16 weeks under light sedation with Zoletil (tiletamine 10 mg/kg, zolazepam 10 mg/kg; Virbac, Peakhurst, NSW, Australia). Measurements were performed using an MLT1010 Piezo-Electric Pulse Transducer (ADInstruments, Bella Vista, NSW, Australia) and an inflatable tail-cuff connected to an MLT844 Physiological Pressure Transducer (ADInstruments) connected to a PowerLab data acquisition unit (ADInstruments) [21].

Dual-energy X-ray absorptiometry (DXA) was performed on rats fed *G. humilis* rind at the end of the 16-week study period using a Norland XR46 DXA instrument (Norland Corp., Fort Atkinson, WI, USA). Rats were anesthetized by intraperitoneal injection of Zoletil (tiletamine 10 mg/kg and zolazepam 10 mg/kg; Virbac) [21].

The isolated Langendorff heart preparation was performed to assess left ventricular function of the rats in all groups [21]. Lethabarb (pentobarbitone sodium, 100 mg/kg; Virbac) administered intraperitoneally was used to induce terminal anesthesia. Following heparin (Sigma-Aldrich Australia) administration (200 IU) into the right femoral vein, blood (~ 5 mL) was collected from the abdominal aorta in heparinized tubes. Isovolumetric ventricular function was measured by inserting a latex balloon catheter into the left ventricle of the isolated heart connected to a Capto SP844 MLT844 physiological pressure and Chart software on a MacLab system (ADInstruments) [21].

Blood was centrifuged at 5000 × *g* for 10 minutes within 30 minutes of collection into heparinized tubes. Plasma was stored at −20 °C before analysis. Plasma concentrations of total cholesterol, triglycerides, non-esterified fatty acids (NEFA), and activities of plasma alanine transaminase (ALT) and aspartate transaminase (AST) were determined using kits and standards supplied by Olympus (Tokyo, Japan) using an AU 400 Olympus analyzer [21]. Plasma catalase activity was measured [23]. An ELISA Rat IL-6 SimpleStep kit (Abcam, Melbourne, VIC, Australia) was used to measure IL-6 concentrations.

The right and left ventricles were separated after perfusion experiments and weighed. After the heart was removed, the liver, and retroperitoneal, epididymal, and omental fat pads were collected and blotted for weighing. Retroperitoneal, epididymal, and omental fat pads were calculated together as total abdominal fat. Organ weights were normalized relative to the tibial length at the time of their removal (in mg/mm).

Approximately 5–7 minutes after euthanasia, heart and liver portions were collected and fixed in 10% neutral buffered formalin for 3 days. The samples were then dehydrated and embedded in paraffin wax. Thin sections (~5 µm) of the left ventricle and liver were cut and stained with hematoxylin and eosin. Stained sections were examined using an EVOS FL Color Imaging System (version 1.4 (Rev 26059); Advanced Microscopy Group, Bothwell, WA, USA) to observe infiltration of inflammatory cells in the liver and heart and for determining fat vacuoles in liver [21]. Heart sections were also stained with picrosirius red staining to study collagen distribution in the left ventricle [21]. The extent of collagen deposition in heart tissues was observed using BX53 Olympus microscope (Olympus Australia, Macquarie Park, NSW, Australia).

### 2.4. Statistical Analysis

All data are presented as mean ± standard error of the mean (SEM). Data from C, CAP, CAR, H, HAP, and HAR groups were tested by two-way analysis of variance (ANOVA). When interaction and/or the main effects were significant, means were compared using Newman-Keuls multiple comparison *post hoc* test. *p*-value of <0.05 was considered as statistically significant. All statistical analyses were performed using Prism version 6.00 for Windows (GraphPad Software, San Diego, CA, USA).

## 3. Results

### 3.1. Weight and Phytochemical Analysis of G. humilis

**Figure 1:**
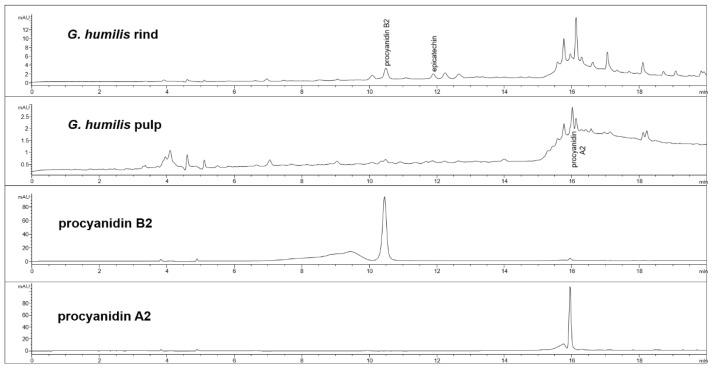
High-performance liquid chromatography (HPLC) chromatograms from *Garcinia humilis* rind and pulp for procyanidin analysis at 280 nm.

**Figure 2:**
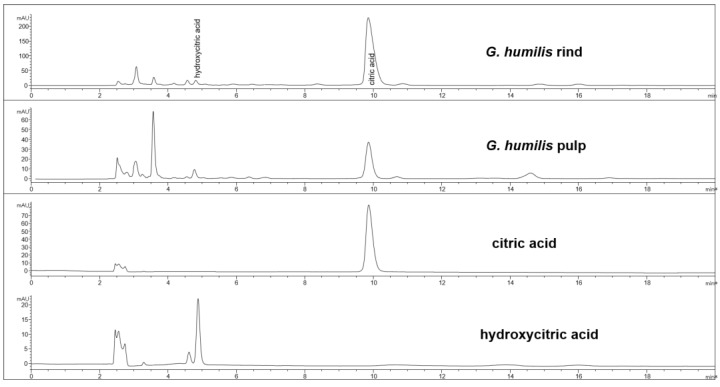
HPLC chromatogram from *G. humilis* rind and pulp, and citric and hydroxycitric acids at 210 nm.

The fruit weighed 44.6 ± 2.7 g with wet weights of the rind, pulp, and seed as 18.95 ± 0.81 g, 14.86 ± 1.61 g and 10.85 ± 0.63 g, respectively. The weight-loss upon drying of the rind, pulp, and seed was 71%, 82%, and 29%, respectively. The rind and seed constituted about 66% of the total fruit weight. Phytochemical analysis of the fruits of *G. humilis* showed the presence of procyanidins (Figure 1), and citric acid (Figure 2), in both the rind and pulp. The rind contained more compounds than the pulp with 6.1% w/w flavanols (epicatechin (1.005 % w/w), catechin (0.099% w/w) and procyanidins (procyanidin B2 (4.898% w/w) and procyanidin A2 (0.043% w/w)) in the rind powder compared to 0.32% w/w flavanols (epicatechin (0.031% w/w), catechin (0.015% w/w), procyanidin B2 (0.274% w/w)) and a very low concentration of procyanidin A2 in the pulp powder. The predominant organic acid in the fruit was citric acid (Figure 2), rather than hydroxycitric acid as reported in many *Garcinia* species [24]. The citric acid content was 12.14% w/w and 1.44% w/w in rind powder and pulp powder, respectively, with very low concentrations of hydroxycitric acid.

### 3.2. Diet Intake and Body Composition

H rats consumed less food than C rats (Table 1). Hence, the intake of procyanidins, flavonoids, and citric acid were higher in CAR than in HAR, and in CAP than in HAP (Table 1). Neither *G. humilis* rind nor pulp supplementation changed food or water intakes (Table 1). There were no changes in the intake of water across groups (Table 1). Although the food intakes in H rats were lower than C rats, the mean energy intake was higher in H rats than in C rats (Table 1). Prolonged H diet intake for 16 weeks increased abdominal circumference, total body fat mass, and abdominal fat pads.

H rats had higher total body fat mass compared to C rats (Table 1). There were no changes in total abdominal fat mass in H, HAP, and HAR rats (Table 1). As the rind intervention showed some effects on cardiovascular parameters, fat and lean mass values were recorded by DXA. However, there was no difference in fat and lean mass between H and HAR or between C and CAR rats. The visceral indices in H rats were higher than C rats (Table 1). There were lower body weight gains in CAR and HAR rats but not in CAP and HAP rats (Table 1). Lower feed conversion efficiency was observed in CAR rats but not in HAR rats. Despite these changes, there were no changes in the final body weights between C, CAP, and CAR, or between H, HAP, and HAR rats (Table 1).

### 3.3. Plasma Biochemistry and Oral Glucose Tolerance

The concentrations of triglycerides and NEFA were higher in H rats compared to C rats (Table 1). However, there were no differences in total cholesterol and NEFA between control and treatment rats (Table 1). Plasma triglyceride concentrations were reduced in HAR rats compared to H rats, but not in HAP rats. Fasting blood glucose concentrations in H rats were higher than in C rats. Overall, there was no improvement in glucose tolerance in HAP and HAR rats (Table 1). Plasma catalase activity was unchanged between H groups. Plasma IL-6 concentrations were below detection.

### 3.4. Cardiovascular Structure and Function

**Figure 3:**
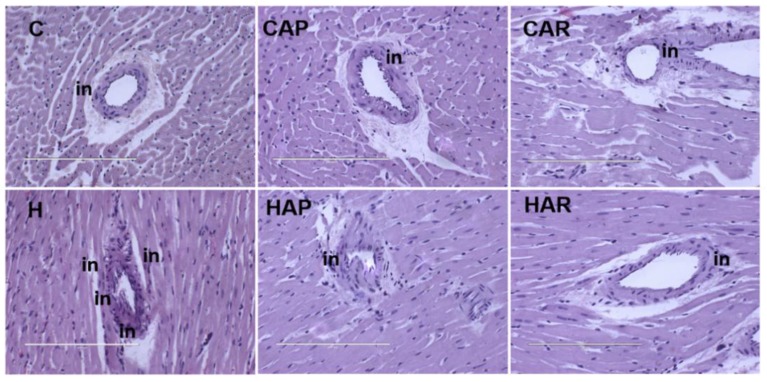
Hematoxylin and eosin staining of left ventricles of heart (magnification ×20; scale bar = 200 µm) showing inflammatory cells (indicated as “in”) as dark spots outside the myocytes in rats fed corn starch diet (C), corn starch diet + achacha pulp (CAP), corn starch diet + achacha rind (CAR), high-carbohydrate, high-fat diet (H), high-carbohydrate, high-fat diet + achacha pulp (HAP), and high-carbohydrate, high-fat diet + achacha rind (HAR).

**Figure 4:**
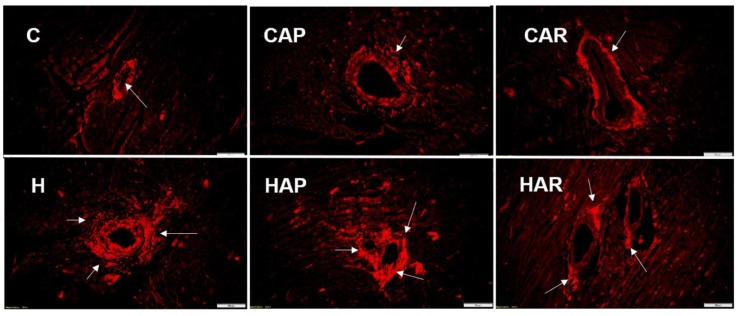
Picrosirius red staining of left ventricular perivascular collagen deposition (magnification ×20; scale bar = 100 µm) showing fibrosis (indicated by arrows) in rats fed corn starch diet (C), corn starch diet + achacha pulp (CAP), corn starch diet + achacha rind (CAR), high-carbohydrate, high-fat diet (H), high-carbohydrate, high-fat diet + achacha pulp (HAP), and high-carbohydrate, high-fat diet + achacha rind (HAR).

H and HAP rats had higher systolic blood pressures compared to C, CAP, CAR, and HAR rats, which were not different (Table 1). In the isolated Langendorff heart preparation, left ventricular diastolic stiffness was higher in H rats compared to HAP, HAR, C, CAP, and CAR rats (Table 1). The left ventricular wet weight (with septum) and the right ventricular wet weight did not differ between the groups. The left ventricle of the hearts from H rats showed increased infiltration of inflammatory cells and collagen deposition compared to C rats, but reduced inflammatory cells were observed in HAR but not HAP rats compared to H rats (Figure 3 and Figure 4).

### 3.5. Liver Structure and Function

**Figure 5:**
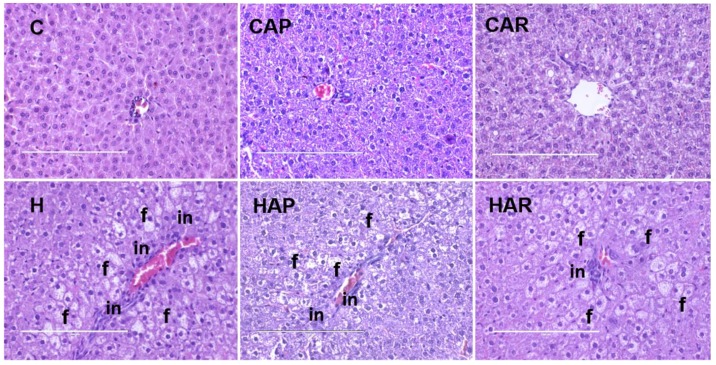
Hematoxylin and eosin staining of hepatocytes (magnification ×20; Scale bar = 200 µm) showing inflammatory cells (marked as “in”) and hepatocytes with fat vacuoles (marked as “f”) in rats fed corn starch diet (C), corn starch diet + achacha pulp (CAP), corn starch diet + achacha rind (CAR), high-carbohydrate, high-fat diet (H), high-carbohydrate, high-fat diet + achacha pulp (HAP), and high-carbohydrate, high-fat diet + achacha rind (HAR).

The normalized liver weight was higher in H rats compared to C rats, and higher in H and HAP rats compared to HAR rats (Table 1). There was an increased infiltration of inflammatory cells and enhanced presence of fat droplets in H rats compared to C rats (Figure 5). However, there was a slight reduction in inflammatory cell infiltration in HAR rats compared to H and HAP rats. Plasma liver enzyme analysis showed increased activity of AST in H and HAP rats compared to HAR, C, CAP, and CAR rats (Table 1). The ALT activity was lower in C, CAP, CAR, and HAR rats compared to H and HAP rats (Table 1).

## 4. Discussion

The pulp of Australian-grown achacha (*G. humilis*) is enjoyed as a tasty fruit [6], similar to reports for *G. humilis* fruit grown in South America [3,4]. The rind and seed of the Australian achacha comprises about 60% of the fruit, similar to other tropical fruits such as durian, mangosteen, and jackfruit, which have seeds and rind comprising more than 50% of the total fruit [24]. The major bioactive constituents of the rind of *G. humilis* were the B-type procyanidins and citric acid. Procyanidins are mainly found in fruits [25,26]; *Garcinia* species such as *G. mangostana* [27,28], *G. brasiliensis* [29], and *G. multiflora* [30] also contain procyanidins. At the dose of rind used in this study, the average dose of procyanidins of around 40–60 mg/kg/day translates to about 500 mg/day in a 70 kg adult human according to surface area comparisons between rats and humans [31]. An intake of 500 mg/day would require the consumption of about 10 g of dried *G. humilis* rind powder per day, which is equivalent to about 35 g of fresh rind, or approximately two fruits.

In rats, decreases in blood pressure were shown at much lower procyanidin doses than in this study at 10 mg/kg/day [32,33], which would translate to about 120 mg procyanidins from about 2 g rind powder or 7 g fresh achacha rind per day, or around 40% of one fruit, in humans. As the rind is not normally used as a food, novel products would be needed, such as the addition of the rind to different dishes including desserts or savory recipes, or as a drink [4,6]. Further, Australian achacha contained a high concentration of citric acid, rather than hydroxycitric acid as in other *Garcinia* species [11,34]. Previous studies have shown the presence of 10–30% citric acid in *G. cambogia* [35]. Dried achacha rind contained 121 mg/g of citric acid, a similar amount to 2.5 mL of fresh lemon juice [36].

The effects of *G. humilis* on metabolic syndrome are unknown, although *Garcinia* fruits have antibacterial, antiviral, antiprotozoal, antiinflammatory, antimicrobial, anticancer, and antiimmunosuppressive properties [1,12]. In a rat model that mimics human metabolic syndrome, our results showed that *G. humilis* rind, but not pulp, improved cardiovascular structure and function, liver structure and function, and plasma triglyceride concentrations with no changes in obesity or other metabolic parameters.

Based on our results, the most likely phytochemical responsible for the cardioprotective effects of *G. humilis* rind was procyanidin B2 due to its abundance in the rind rather than the pulp, and based on reported studies. However, the interactions of different phytochemicals present in the rind, particularly procyanidin B2 and citric acid, could produce synergistic effects responsible for the observed cardiovascular improvements in this study. The biological effects of procyanidins have been attributed to the absorption of colonic breakdown products such as phenolic acids and valero-lactones [37]. Procyanidin B2 was metabolized by human fecal microbiota yielding 5-(2’,4’-dihydroxy) phenyl-2-ene valeric acid and 5-(3’,4’-dihydroxyphenyl) valeric acid after *in vitro* incubation of procyanidin B2 with human fecal microbiota [38]. The extent of bacterial degradation in the gut diminished with the increase in monomeric units, with the yield of phenolic acids in rat gut decreasing from 7% for dimers to 0.7% and 0.5% for trimers and polymers, respectively [39]. In addition, the procyanidin B dimer present in the rind powder can be absorbed from the rat small intestine [40], and was present in human plasma 30 minutes after consumption of procyanidin-containing foods [41].

Metabolic syndrome includes physiological changes such as central obesity, insulin resistance, hypertension, impaired glucose tolerance, and dyslipidemia that enhance the risk of developing cardiovascular disease, type 2 diabetes, and non-alcoholic fatty liver disease [42]. Administration of 50, 100, 300, and 600 mg/kg of cocoa extract rich in procyanidin B2 in water reduced blood pressure in male spontaneously hypertensive rats [43]. In addition, oral gavage administration of 50 mg/kg/day of procyanidin B2 for 4 weeks reduced blood pressure in L-NAME–induced hypertensive Sprague Dawley rats [44]. Moreover, the addition of 10 mg/kg of procyanidin B2 in a hypercaloric diet reduced blood pressure in male Wistar rats [45]. Thus, doses of procyanidins even lower than in this study were able to decrease blood pressure in hypertensive rats. In general, there is still limited information regarding the effects of procyanidin B2 on systolic blood pressure in rats as most literature did not specifically mention procyanidin types in their studies.

Several mechanisms have been suggested to explain the action of procyanidins in reducing blood pressure. Procyanidins may inhibit angiotensin-converting enzyme, which could be related to the number of hydroxyl groups on the procyanidins [46]. In addition, procyanidins also could increase mRNA expression of Kruppel-like factor 2 (KLF2) which is involved in the regulation of endothelial gene expression in response to laminar flow [47]. In addition, procyanidin B2 showed protective effect in vascular smooth muscle cells by its antioxidative effects [48]. Both procyanidin B2 and epicatechin showed cardioprotection by stimulating substrate-driven mitochondrial respiration and decreasing reactive oxygen species production in isolated rat heart mitochondria [49].

Further, procyanidin B2 reduced oxidative and inflammatory responses in *in vivo* rodent models and in cell culture models. Procyanidin B2 pretreatment (25, 50, and 100 mg/kg of body weight) showed antiinflammatory responses by inhibiting mRNA and protein expression of hepatic COX-2, iNOS, TNF-α, IL-1β, and translocation of NF-κB p65 from cytosol to the nuclear fraction in mouse hepatocytes [50]. In addition, procyanidin B2 administration showed antioxidant actions as decreased malondialdehyde concentrations and increased activities of glutathione peroxidase, superoxide dismutase, and catalase in mice [50] and in hypertensive rats (50 mg/kg body weight of procyanidin B2) [44]. However, the inflammation and oxidative stress in metabolic syndrome is described as a chronic low-grade state, which may be the reason for the unchanged plasma catalase activities in H rats, and the undetectable plasma IL-6 concentrations.

Dietary supplementation of 0.002% of procyanidin B2 in streptozotocin-induced diabetic rats showed antioxidant effects by delaying the formation and progression of cataracts in the rat model through the inhibition of advanced glycation end products [51]. In a rat model of cerebral ischemia, intragastric administration of procyanidin B2 (40 mg/kg) reduced mitochondrial depolarization and reactive oxygen species in the ipsilateral ischemic area. The treatment also reduced malondialdehyde concentrations and promoted antioxidant enzyme activities (catalase and superoxide dismutase) compared to the vehicle-treated group, which were related to the activation of Nrf2 protein that upregulates antioxidant defences [52]. Procyanidin B2 supplementation also reduced 4-hydroxynonenal which is a measure of lipid peroxidation by reactive oxygen species [53] and advanced glycation end products [54].

In general, procyanidin B2 could suppress oxidative stress and inflammation through prevention of lipid peroxidation, inhibition of NF-κB and MAPK pathways, activation of Nrf2 pathways, inhibition of glutathione consumption, blockade of JNK activation pathways, decreased expression of TNF-α, IL-1β, and iNOS, modulation of arachidonic acid pathways by inhibition of COX-2 gene, activation of AMPK-SIRT1-PGC1-α axis, inhibition of NLRP3 inflammasome activation, and suppression of reactive oxygen species generation [50,55,56,57,58,59,60,61].

In our study, the dose of procyanidins (42 mg/kg/day) could explain the absence of body weight reduction and metabolic effects compared to previous studies using higher doses [62,63]. Similarly, a study using administration of 50 mg/kg/day of procyanidin B2 did not reduce body weight in hypertensive rats [44]. As in our study, no differences were shown in serum NEFA concentrations between high-fat and groups treated with epicatechin (10 mg/kg), catechin (10 mg/kg), or procyanidin B2 (10 mg/kg) except those treated with the combination as cocoa powder (100 mg/kg) [64]. However, administration of 30 mg/kg/day of grape seed procyanidin B2 in diabetic *db*/*db* mice reduced body weight [53].

In this study, we found a reduction in plasma triglycerides concentrations and activities of ALT and AST, and decreased liver weight of high-carbohydrate, high-fat diet-fed rats with *G. humilis* rind. Pretreatment with procyanidin B2 also prevented the increase of serum ALT and AST in CCl_4_-induced hepatic injury in mice, which was linked to its antiapoptotic properties in hepatocytes [50]. Further, administration of 30 mg/kg/day of grape seed procyanidin B2 decreased serum triglycerides, total cholesterol, and free fatty acids concentrations [53]. In addition, the study noted reductions in hepatic lipid droplet accumulation and content. The changes were associated with the increase in phosphorylation activities of AMPK and ACC enzymes with increased concentration of CPT1 which are required for oxidation of fatty acids [53].

In this study, we did not find any improvement in the oral glucose tolerance test after supplementation with a low procyanidin dose of *G. humilis* rind. Administration of 30 mg/kg/day of procyanidin B2 did not reduce fasting blood glucose in diabetic mice [54]. However, the treatment reduced serum insulin concentrations, HOMA-IR values, and the pancreatic expression of IL-1β and NLRP3 indicating the antiinflammatory activities of procyanidin B2 [54].

Citric acid present in the rind of *G. humilis* fruits could increase the responses of procyanidin B2. Citric acid is naturally found in citrus fruits and usually used as a food additive to give a sour taste to foods and drinks [36]. Citric acid showed hypotensive effects in normotensive mice, producing 23% and 71% reductions in mean arterial blood pressure at doses of 3 mg/kg and 15 mg/kg, respectively [65]; in our study, citric acid was administered at 104 mg/kg/day in HAR rats (Table 1). In addition, an investigation on the consumption of lemon juice, walking, and blood pressures in 33–77 year-old Japanese women showed that blood citric acid concentrations negatively correlated with the changes in blood pressure [66]. Citric acid may promote the absorption of calcium and magnesium that could affect blood pressure [67,68,69]. However, the relationship between citric acid intake and its effect on metabolic syndrome is still inconclusive. In contrast to other *Garcinia* species, we found very low concentrations of hydroxycitric acid in our sample. Hydroxycitric acid was shown to have an antiobesity effect in rats when supplemented in the diet [70,71].

Analyses of the pulp of *G. humilis* showed much lower concentrations of procyanidins and citric acid than the rind, and very low hydroxycitric acid concentrations. Treatment with the pulp did not change metabolic syndrome parameters in our study, suggesting that the doses of these phytochemicals were too low to be effective. Although there were no observed changes in blood pressure, the pulp reduced the diastolic stiffness of the heart in H-diet rats, indicating potential cardioprotective properties. The pulp of the fruit is usually eaten as a dessert fruit and this suggests the presence of simple sugars [3] that may be useful as a source of energy or micronutrients such as vitamins or minerals.

Although hydroxycitric acid was present in very low concentrations in *G. humilis*, citric acid as the main organic acid could interact with procyanidins and other phytochemicals to improve the cardiovascular parameters seen in our rat model. As clinical studies on the effect of procyanidins were mainly performed with grape or cocoa products also containing flavanols but not citric acid, we propose that the combination of procyanidins with citric acid in achacha fruit rind should be tested in overweight humans to attenuate the cardiovascular and liver signs of metabolic syndrome.

## 5. Conclusions

This study reports the positive responses to *G. humilis* rind powder containing procyanidin B2 and citric acid in improving the cardiovascular parameters in rats fed a high-carbohydrate, high-fat diet. We recommend that further *in vivo* studies of *G. humilis* are performed to explore its therapeutic potential.

## Figures and Tables

**Table 1:** Effects of *G. humilis* on abdominal fat content, metabolic, and physiological parameters.

Variables	C	CAP	CAR	H	HAP	HAR	*p* value		
							Diet	Treatment	Interaction
Initial body weight (0 week), g	340 ± 3 ^a^	341 ± 1 ^a^	339 ± 1 ^a^	338 ± 1 ^a^	338 ± 2 ^a^	339 ± 1 ^a^	0.09	0.86	0.44
Midpoint body weight (8 week), g	361 ± 9 ^b^	347 ± 5 ^b^	363 ± 6 ^b^	437 ± 9 ^a^	414 ± 7 ^a^	438 ± 8 ^a^	<0.0001	0.02	0.82
Final body weight (16 week), g	403 ± 10 ^b^	380 ± 7 ^b^	385 ± 7 ^b^	524 ± 14 ^a^	504 ± 8 ^a^	512 ± 12 ^a^	<0.0001	0.11	0.97
Food intake, g/day	38.7 ± 2.1 ^a^	35.1 ± 2.0 ^ab^	37.6 ± 1.9 ^a^	27.8 ± 2.5 ^b^	27.2 ± 2.2 ^b^	28.6 ± 2.8 ^b^	<0.0001	0.59	0.80
Water intake, g/day	33.6 ± 2.5 ^a^	27.3 ± 2.9 ^a^	30.1 ± 2.7 ^a^	29.5 ± 2.1 ^a^	28.1 ± 2.2 ^a^	31.4 ± 2.1 ^a^	0.77	0.36	0.57
Flavonoids intake, mg/kg/day	-	4.5 ± 0.2 ^c^	67.6 ± 3.0 ^a^	-	3.5 ± 0.6 ^c^	51.4 ± 2.9 ^b^	0.0002	<0.0001	<0.0001
Procyanidin B2 intake, mg/kg/day	-	3.8 ± 0.2 ^c^	55.2 ± 2.5 ^a^	-	2.9 ± 0.5 ^c^	42.0 ± 2.4 ^b^	0.0003	<0.0001	<0.0001
Citric acid intake, mg/kg/day	-	20.2 ± 1.0 ^c^	136.9 ± 6.1 ^a^	-	15.6 ± 2.7 ^c^	104. 2 ± 5.9 ^b^	0.0001	<0.0001	<0.0001
Energy intake, kJ/day	434 ± 41 ^b^	401 ± 28 ^b^	423 ± 21 ^b^	636 ± 47 ^a^	595.8 ± 43 ^a^	642 ± 50 ^a^	<0.0001	0.59	0.95
Feed conversion efficiency, g/kJ	0.09 ± 0.01 ^bc^	0.09 ± 0.01 ^bc^	0.06 ± 0.00 ^c^	0.14 ± 0. 01 ^a^	0.15 ± 0.01 ^a^	0.11 ± 0.01 ^b^	<0.0001	0.0004	0.19
Body weight gained (8–16 week), %	11.8 ± 0.9 ^c^	9.63 ± 0.9 ^cd^	6.77 ± 0.7 ^d^	19.8 ± 1.3 ^a^	22.0 ± 1.1 ^a^	16.8 ± 1.4 ^b^	<0.0001	0.0004	0.14
Abdominal circumference, cm	19.0 ± 0.3 ^b^	18.5 ± 0.1 ^b^	18.9 ± 0.2 ^b^	21.6 ± 0.3 ^a^	21.4 ± 0.4 ^a^	21.0 ± 0.3 ^a^	<0.0001	0.37	0.22
Body mass index, kg/m^2^	6.28 ± 0.13 ^b^	6.32 ± 0.21 ^b^	6.51 ± 0.13 ^b^	8.04 ± 0.21 ^a^	7.61 ± 0.18 ^a^	7.93 ± 0.21 ^a^	<0.0001	0.36	0.43
Retroperitoneal fat, mg/mm *	276 ± 24 ^b^	252 ± 11 ^b^	234 ± 16 ^b^	565 ± 47 ^a^	517± 44 ^a^	525 ± 36 ^a^	<0.0001	0.37	0.90
Epididymal fat, mg/mm *	99 ± 9 ^b^	71± 8 ^b^	75 ± 9 ^b^	180 ± 41 ^a^	193 ± 19 ^a^	143 ± 16 ^a^	<0.0001	0.13	0.21
Omental fat, mg/mm *	160 ± 12 ^b^	122 ± 10 ^b^	118 ± 9 ^b^	255 ± 21 ^a^	256 ± 10 ^a^	231 ± 15 ^a^	<0.0001	0.058	0.35
Total abdominal fat, mg/mm *	536 ± 39 ^b^	445 ± 24 ^b^	427 ± 26 ^b^	1000 ± 85 ^a^	965 ± 70 ^a^	902 ± 62 ^a^	<0.0001	0.18	0.87
Visceral adiposity index, %	6.23 ± 0.38 ^b^	4.92 ± 0.22 ^b^	5.21 ± 0.29 ^b^	9.11 ± 0.47 ^a^	8.81 ± 0.53 ^a^	8.76 ± 0.40 ^a^	<0.0001	0.09	0.43
Liver weight, mg/mm *	234 ± 8 ^cd^	246 ± 7 ^c^	214 ± 4 ^d^	355 ± 12 ^a^	357 ± 10 ^a^	320 ± 10 ^b^	<0.0001	0.0008	0.68
**Plasma biochemistry**									
Basal blood glucose (16 week), mmol/L	3.9 ± 0.2 ^b^	3.8 ± 0.2 ^b^	3.9 ± 0.1 ^b^	4.7 ± 0.1 ^a^	4.6 ± 0.2 ^a^	4.7 ± 0.1 ^a^	<0.0001	0.76	0.99
Area under the curve (16 week), mmol/L·minute	647 ± 26 ^b^	652 ± 18 ^b^	623 ± 18 ^b^	768 ± 30 ^a^	752 ± 15 ^a^	743 ± 17 ^a^	<0.0001	0.80	0.81
ALT, U/L	29.6 ± 2.6 ^b^	31.3 ± 2.9 ^bc^	31.0 ± 1.0 ^bc^	44.0 ± 3.4 ^a^	45.5 ± 3.8 ^a^	36.1 ± 2.0 ^b^	<0.0001	0.18	0.14
AST, U/L	73.6 ± 3.4 ^b^	81.4± 6.1 ^b^	74.0 ± 2.0 ^b^	104.6 ± 6.2 ^a^	92.3 ± 5.9 ^ab^	85.3 ± 3.0 ^b^	<0.0001	0.10	0.046
Total cholesterol, mmol/L	1.40 ± 0.1 ^a^	1.53 ± 0.05 ^a^	1.45 ± 0.1 ^a^	1.60 ± 0.1 ^a^	1.59 ± 0.05 ^a^	1.60 ± 0.05 ^a^	0.0409	0.75	0.68
Triglyceride, mmol/L	0.62 ± 0.1 ^d^	0.74 ± 0.1 ^dc^	0.5 ± 0.1 ^d^	2.00 ± 0.2 ^a^	1.87 ± 0.28 ^a^	1.27 ± 0.2 ^bc^	<0.0001	0.028	0.23
NEFA, mmol/L	1.27 ± 0.2 ^b^	1.23 ± 0.18 ^b^	1.5 ± 0.2 ^b^	3.74 ± 0.4 ^a^	3.73 ± 0.25 ^a^	3.99 ± 0.3 ^a^	<0.0001	0.55	0.98
Catalase activity, kU/L	43.7 ± 7.6 ^b^	46.7 ± 6.7 ^b^	36.9 ± 2.3 ^b^	51.7 ± 5.58 ^a^	53.6 ± 7.7 ^a^	60.0 ± 5.86 ^a^	0.0166	0.9224	0.361
**Cardiovascular variables**									
Systolic blood pressure (16 week), mmHg	127 ± 2 ^b^	126 ± 3 ^b^	129 ± 2 ^b^	143 ± 3 ^a^	141 ± 5 ^a^	130 ± 3 ^b^	<0.0001	0.16	0.029
LV + Septum, mg/mm *	22.8 ± 1.0 ^a^	22.3 ± 1.1 ^a^	21.9 ± 0.6 ^a^	23.0 ± 0.9 ^a^	23.4 ± 0.6 ^a^	22.8 ± 0.6 ^a^	0.28	0.76	0.85
Right ventricle, mg/mm *	4.73 ± 0.31 ^a^	5.01 ± 0.24 ^a^	4.95 ± 0.31 ^a^	5.57 ± 0.39 ^a^	5.61 ± 0.56 ^a^	4.76 ± 0.32 ^a^	0.17	0.46	0.35
Diastolic stiffness constant (*κ*)	22.1 ± 0.4 ^c^	21.6 ± 0.8 ^c^	20.9 ± 0.3 ^c^	27.0 ± 0.8 ^a^	24.7 ± 0.9 ^b^	22.6 ± 0.6 ^c^	<0.0001	0.0004	0.034

Values are expressed as mean *±* SEM, *n* = 8–12. Means with different superscripts differ, *p* < 0.05. LV, left ventricle; AST, aspartate transaminase; ALT, alanine transaminase; NEFA, non-esterified fatty acids; C, corn starch diet fed rats; CAP, corn-starch diet with achacha pulp, CAR, corn starch diet with achacha rind; H, high-carbohydrate, high-fat diet-fed rats; HAP, high-carbohydrate, high-fat diet with achacha pulp; HAR, high-carbohydrate, high-fat diet with achacha rind. * Denotes the values were normalized against tibial length and given as the tissue weight in mg/mm.
